# Redox crosstalk at endoplasmic reticulum (ER) membrane contact sites (MCS) uses toxic waste to deliver messages

**DOI:** 10.1038/s41419-017-0033-4

**Published:** 2018-02-28

**Authors:** Edgar Djaha Yoboue, Roberto Sitia, Thomas Simmen

**Affiliations:** 1 Protein Transport and Secretion Unit, Division of Genetics and Cell Biology, IRCCS Ospedale San Raffaele, Università Vita-Salute San Raffaele, Milan, Italy; 2 Department of Cell Biology, University of Alberta, Edmonton, AB Canada T6G2H7

## Abstract

Many cellular redox reactions housed within mitochondria, peroxisomes and the endoplasmic reticulum (ER) generate hydrogen peroxide (H_2_O_2_) and other reactive oxygen species (ROS). The contribution of each organelle to the total cellular ROS production is considerable, but varies between cell types and also over time. Redox-regulatory enzymes are thought to assemble at a “redox triangle” formed by mitochondria, peroxisomes and the ER, assembling “redoxosomes” that sense ROS accumulations and redox imbalances. The redoxosome enzymes use ROS, potentially toxic by-products made by some redoxosome members themselves, to transmit inter-compartmental signals via chemical modifications of downstream proteins and lipids. Interestingly, important components of the redoxosome are ER chaperones and oxidoreductases, identifying ER oxidative protein folding as a key ROS producer and controller of the tri-organellar membrane contact sites (MCS) formed at the redox triangle. At these MCS, ROS accumulations could directly facilitate inter-organellar signal transmission, using ROS transporters. In addition, ROS influence the flux of Ca^2+^ ions, since many Ca^2+^ handling proteins, including inositol 1,4,5 trisphosphate receptors (IP_3_Rs), SERCA pumps or regulators of the mitochondrial Ca^2+^ uniporter (MCU) are redox-sensitive. Fine-tuning of these redox and ion signaling pathways might be difficult in older organisms, suggesting a dysfunctional redox triangle may accompany the aging process.

## Facts

- The ER forms membrane contact sites (MCS) with mitochondria (mitochondria-associated membrane, MAM), as well as peroxisomes
- Redox-sensitive proteins localize to physical contacts between the endoplasmic reticulum (ER) and mitochondria, known as mitochondria-ER contacts (MERCs)
- Redox signaling takes place at the “redox triangle” formed by mitochondria, peroxisomes and the ER
- Given the hormetic properties of reactive oxygen species (ROS, essential or toxic, depending on their concentrations) their trans-organellar transport and diffusion are likely regulated

## Open Questions

- Does a multimeric, mitochondria, peroxisome and ER-associated protein complex exist that forms a “redoxosome” whose composition differs from cell to cell?
- Does protein folding and/or the unfolded protein response (UPR) influence the redox state of mitochondria and peroxisomes, as well as their Ca^2+^ handling machinery?
- Are there ROS conducting channels at MCS?
- What are the consequences of a dysfunctional redoxosome during aging?

## Introduction

Reduction and oxidation reactions, summarized as redox, are reactions where there is gain (reduction) or loss (oxidation) of electron(s) by atoms of the reacting species. This plethora of chemical reactions governs virtually all aspects of life. Our essay focuses on the redox interplay between three organelles that produce reactive oxygen species (ROS), mitochondria, peroxisomes, and the endoplasmic reticulum (ER)^1^. A common redox background of these organelles is reflected on the cell biological level by physical and functional contacts between the three that defy the classic textbook notion of stand-alone organelles with well-defined single functions^2^. These inter-organellar physical connections allow for the exchange of material in the form of metabolites, protein and lipid material, and ROS that influence the cellular redox^3^. Although functional connections were initially hard to detect^4^, the first descriptions of organellar apposition between the three organelles were made during the early phases of electron microscopy studies in the mid 20th century, for example between the ER and mitochondria^5,6^, or between the ER, mitochondria and peroxisomes^7^. Amongst these types of membrane contact sites (MCS), those between mitochondria and ER are probably the best studied. These contacts are biochemically known as mitochondria-associated membranes (MAMs)^8,9^, where redox-regulatory proteins are enriched^10,11^.

Prominent MAM-localized regulators of ER-mitochondria crosstalk are calnexin^12,13^, Ero1α^14,15^ or selenon/SEPN1^16^. These chaperones and oxidoreductases, some of which produce ROS themselves, bind to ER Ca^2+^ handling proteins and determine ER-mitochondria Ca^2+^ flux and, thus, mitochondrial metabolism via redox-dependent interactions^17^. Therefore, the mechanism of ER oxidative protein folding moonlights as a regulator of mitochondria metabolism^18^, as had been proposed in 1958 by Silvio and Anna Fiala who had discovered that the extent of mitochondrial respiration is proportional to the extent of ER protein production^19^.

However, the cellular array of ROS-dependent, redox-sensitive MCS is not limited to the MAM: While the ER may be the most significant ROS producer in some cell types, as assayed by fluorescent probes^20^, and mitochondria are well known producers of ROS via oxidative phosphorylation^21^, peroxisomes are another source of ROS^22^ (summarized in Table 1). It is therefore expected that these organelles participate in redox exchanges with the ER and mitochondria. This is the case, for instance, during the metabolism of etherphospholipids that requires the transport of lipid intermediates from peroxisomes to the ER, where they undergo enzymatic reduction^23^. In the case of mitochondria and peroxisomes, redox-controlling relationships occur for instance during beta-oxidation of fatty acids. While mitochondrial beta-oxidation produces CO_2_ and H_2_O as end products^24^, peroxisomal beta-oxidation of fatty acids is incomplete and produces nicotinamide adenine dinucleotide (NADH) that must be shuttled to mitochondria for complete oxidation^1^. Conversely, NAD+ is likely shuttled into peroxisomes by SLC25A17 in mammalian cells^25^. A related pathway can use the yeast peroxisomal matrix protein Mdh3p, which converts oxaloacetate to malate through the oxidation of NADH^26^, a reaction that is critical for the maintenance of the peroxisomal redox balance^27^. In mammalian cells, a similar shuttle system uses a peroxisomal lactate dehydrogenase that converts pyruvate to lactate, where SLC16A1 shuttles pyruvate in and lactate out^28^. At the moment, it is unclear whether similar shuttling is involved in the metabolism of very long chain fatty acid (VLCFA) degradation products from peroxisomes to mitochondria^29^.

**Table 1:** A list of ROS producing enzymes in the ER, mitochondria, and peroxisomes

Organelle	Protein	Processes associated to ROS production
Mitochondria	NADH:*ubiquinone reductase* (Complex I)	O_2_-generation due to electron leakage during mitochondrial respiratory chain functioning. (Process strongly influenced by mitochondrial parameters)
	*Succinate dehydrogenase* (Complex II)	
	Cytochrome bc1 complex (Complex III)	
	*Pyruvate and α-ketoglutarate dehydrogenases complexes*	Unwanted O_2_-generation through the flavin co-factor of the DLD (*dihydrolipoamide dehydrogenase*) subunit
	*Glycerol-3-phosphate dehydrogenase*	Unwanted O_2_-generation during electrons’ fueling of the mitochondrial respiratory chain
	Electron transfer Flavoprotein (ETF)	
	*Superoxide dismutases* 1 and 2 (SOD1 and SOD2)	H_2_O_2_ production in the mitochondrial matrix (SOD2) and IMS (SOD1) through O_2_-dismutation
Endoplasmic reticulum	ER oxidoreductin(s) (ERo1α and β)	Catalyzed H_2_O_2_ production using O_2_ to initiate disulfide bonds formation during proteins’ folding
	Quiescin sulfhydryl oxidase	
	NADPH oxidase 4 (NOX4)	Sequential production of O_2_ and H_2_O_2_ using NADPH and O_2_
Peroxisomes	*Acyl-coA oxidase(s)* (ACOX1, ACOX2 and ACOX3)	(Peroxisomal fatty acids β-oxidation). Flavine-dependent H_2_O_2_ production
	*Xanthine oxidase* (XO)	H_2_O_2_ and O_2_ production during its catalytic cycle. Involved in processes such as purine metabolism
	*D-amino acid oxidase* (DAO) and *D-aspartate oxidase* (DDO)	H_2_O_2_ production during catalyzed oxidation of D-isomers of aminoacids
	*L-pipecolic acid oxidase* (PIPOX)	H_2_O_2_ production during catalyzed oxidation of pipecolic acid. (involved in lysine degradation)
	*L-α-hydroxyacid oxidase(s)* (HAO1 and HAO2)	H_2_O_2_ production during catalyzed oxidation of glycolic acid
	*Polyamine oxidase* (PAOX)	Involved in the degradation of polyamines such as spermine. H_2_O_2_ during its catalytic process.
	*Urate oxidase* (UO)	H_2_O_2_ production during catalysis of uric acid oxidation. (absent in humans)
	*Superoxide dismutase 1* (SOD1)	H_2_O_2_ production through O_2_dismutation

Although all three organelles are equipped with redox-preserving defense mechanisms, ROS are thought to rapidly diffuse across membranes via certain aquaporins or other specific proteinaceous channels leading to fateful accumulations of these signaling molecules in the proximity of the three organelles^30,31^, as well as potentially lipid droplets^32^. From here, they may influence organellar homeostasis on the redox triangle: for instance, the inhibition of peroxisomal catalase, concomitant with increasing ROS, results in mitochondrial redox imbalance^33^. Moreover, peroxisomal catalase is under the control of the ER unfolded protein transcription factor Xbp-1, suggesting a need for this enzyme during ER stress^34^.

**Fig. 1:**
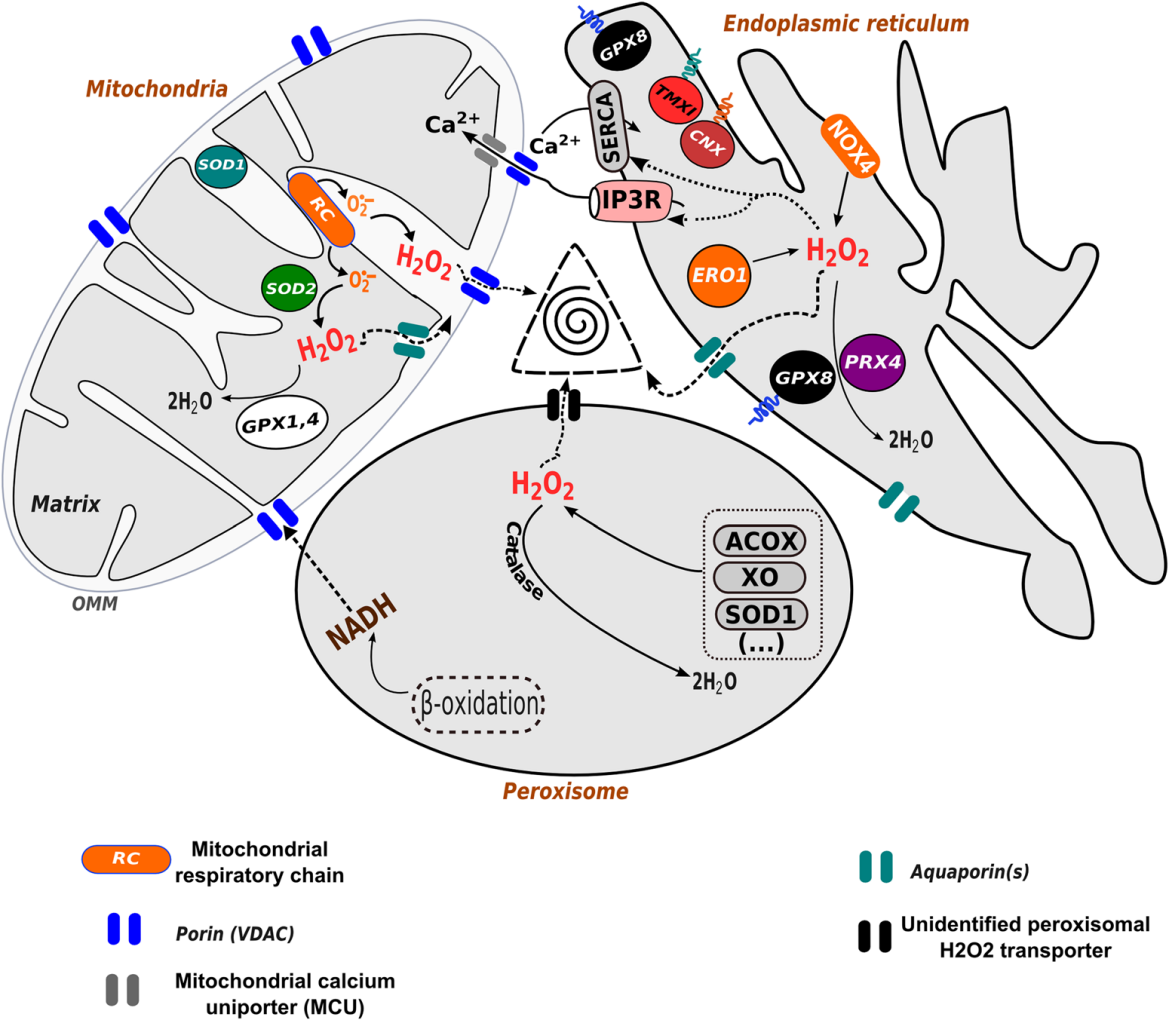
The ER–mitochondria–peroxisome redox triangle The endoplasmic reticulum (ER), mitochondria and peroxisomes are three important redox-sensitive organelles. All three house biochemical reactions that produce reactive oxygen species (ROS), for the list of ROS producers see Table 1. ROS can be released by all three organelles through aquaporins or as of yet unknown proteinaceous channels. Accumulated ROS within the redox triangle affect the functioning of ER–mitochondria Ca^2+^ exchange, oxidative phosphorylation, and especially oxidative protein folding within the ER

Thus, we propose that ternary ER-mitochondria-peroxisome structures form a “redox triangle”, mediated by tethering complexes between the ER and mitochondria^35^, between peroxisomes and mitochondria^36^, as well as between peroxisomes and the ER^37,38^ (summarized in Fig. 1). The redox triangle may become dysfunctional with aging, as shown by decreases of peroxisomal catalase in older cells^39^.

## ROS sources and sinks in mitochondria, peroxisomes and the ER

The powerful oxidant dioxygen or molecular oxygen (O_2_) exhibits a particular chemistry^40,41^, also termed the molecular O_2_ paradox^42^. While O_2_ reduction ultimately can result in its transformation to water, this reduction requires the acceptance of one electron at a time. The O_2_ chemical behavior frequently results in incomplete O_2_ reductions, generating oxidizing species which can (non)catalytically react with biomolecules. Such oxidizing species are ROS, like superoxide anion, hydroxyl radicals, hypochlorous acid and hydrogen peroxide (H_2_O_2_) to name but a few examples.

While basal ROS levels by mitochondria, peroxisomes and the ER are low due to organellar defense systems, redox modifications especially on the mitochondrial respiratory chain can strongly impact on ROS production and can trigger mitochondrial-induced oxidative stress^43,44^. As outlined by ref. 45, the organellar ROS production is dependent on cell and tissue types, as well as oxygen tension. Considering absolute production within the three organelles and their relative permeability for ROS (Table 1), the ER emerges as the relative most important contributor to cytosolic ROS amounts from purified organelles with approximately 60% of ROS derived from this organelle, and the remainder evenly split between mitochondria and peroxisomes^45^. Due to the diverse amounts of ROS scavengers within the three organelles (Table 2), the absolute ROS production amounts are, however, different. When examining perfused liver tissue, the contribution of the ER to total ROS production is negligible, while peroxisomes emerge as a prime ROS production site. In addition to diverse levels of ROS scavengers found within the peroxisomes, mitochondria and the ER, these observations also suggest that the proper formation of contacts between the ER, mitochondria, and peroxisomes could determine the relative cellular ROS contribution of each organelle. Altered cell growth conditions, such as altered protein folding demand, may impact on these findings.

**Table 2:** A list of human ROS scavenging enzymes in the ER, mitochondria and peroxisomes

Organelle	Protein	Processes associated to ROS production
Mitochondria	*Glutathione peroxidase* 1 (GPX1; mitochondrial isoform)	Reduction of H_2_O_2_ in H_2_O using a selenocysteine catalytic residue and reduced glutathione (GSH) as co-factor.
	*Glutathione peroxidase* 4 (GPX4; mitochondrial isoform)	
	Peroxiredoxin 3 (PRDX3/PRX3)	Reduction of H_2_O_2_ in H_2_O using a catalytic cysteine residue. (PRDX5 is also localized in peroxisomes)
	Peroxiredoxin 5 (PRDX5/PRX5)	
	*Superoxide dismutase* 1 (SOD1)	O_2_ dismutation in the mitochondrial matrix (SOD2) and IMS (SOD1) leading to H_2_O_2_ production
	*Superoxide dismutase* 2 (SOD2)	
Endoplasmic reticulum	*Glutathione peroxidase* 7 (GPX7)	Reduction of H_2_O_2_ in H_2_O using cysteine catalytic residues. Contrary to their mitochondrial homologs, they do not use GSH but ER reduced proteins as “co-factors”.
	*Glutathione peroxidase* 8 (GPX8)	
	Peroxiredoxin 4 (PRDX4/PRX4)	Reduction of H_2_O_2_ in H_2_O using a catalytic cysteine residue
Peroxisomes	*Catalase* (CAT)	Hemoprotein catalyzing the reduction of H_2_O_2_ in H_2_O.
	*Superoxide dismutase* 1 (SOD1)	O_2_ dismutation into H_2_O_2_
	*Peroxiredoxin* 5 (PRDX5/PRX5)	Reduction of H_2_O_2_ in H_2_O using a catalytic cysteine residue (also localized in mitochondria)

The most important source of the high amounts of ROS within peroxisomes are the enzymatic activities of oxidases^46^. Amongst these are fatty acid beta-oxidation by acyl-CoA oxidases (ACoX), a group of flavin adenine dinucleotide (FAD)-dependent enzymes under the control of peroxisome-proliferator activated receptors (PPARs) that oxidize long acyl-CoAs^47^. Peroxisomes are equipped with their own defense system that comprises catalase, SOD and Prx5/PMP20^46^, which absorb the majority of the organelle’s ROS, resulting in only minor amounts of cytosolic ROS derived from peroxisomes^45^.

This is not the case for mitochondrial and ER ROS, although these organelles also have their ROS defense systems in the form of peroxiredoxins and other enzymes^48,49^. The main ROS defense systems within mitochondria are superoxide dismutase 1 (SOD1) in the inter-membrane space (IMS) and SOD2 in the mitochondrial matrix. Both enzymes rapidly convert superoxide into H_2_O_2_. In addition, mitochondria also contain glutathione peroxidases, particularly associated to the outer mitochondrial membrane (OMM), where they protect lipids from oxidative modifications^50,51^. Another mitochondrial redox event is the catalyzed formation of disulfide bonds in the IMS. Indeed, contrary to the mitochondrial matrix where the aforementioned antioxidant systems restrict their formation, the yeast oxidoreductases Mia40 and Erv1 catalyze disulfide bonds in the IMS, thus forming a mitochondrial disulfide relay process^52^. Here, CHCHD4, the human homolog of Mia40 interacts with apoptosis-inducing factor (AIF), a chaperone of oxidative phosphorylation^53,54^. This interaction determines CHCHD4 import to the IMS and, therefore, oxidative phosphorylation. The activity of Mia40/CHCDH4 is directly connected to the cytosolic redox, since changes thereof that can be transmitted through VDAC determine its activity^55^.

**Fig. 2:**
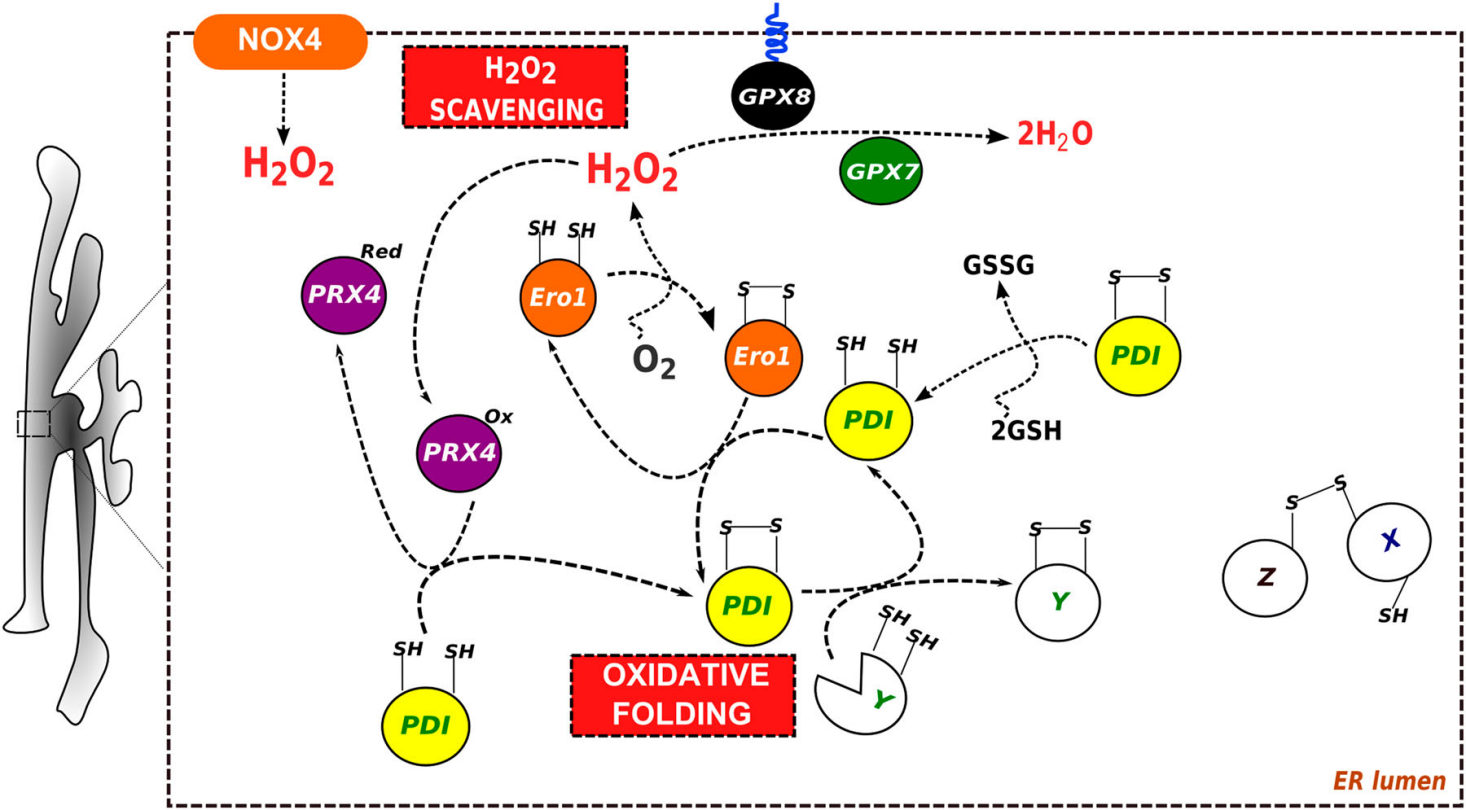
Overview of the ER redox processes The oxidation of Ero1 by O_2_ initiates disulfide relays leading to the insertion of disulfide bonds into proteins as they fold in the ER (here, the protein “Y”) with oxidoreductases of the PDI family playing a key intermediary role (see main text for details). H_2_O_2_ produced during Ero1 oxidation can be scavenged by the peroxidases Gpx7, Gpx8 and Prx4. H_2_O_2_ is also formed in the ER NOX4. Upon H_2_O_2_ scavenging, oxidized Prx4 and Gpx7 transfer a disulfide bond to PDI, contributing to oxidative protein folding. Intermolecular disulfide bonds are also formed into the ER for establishing oligomeric covalent structures or for example retaining misfolded proteins

Inside the ER, ROS are produced from catalytic processes by the oxidoreductase Ero1 (Fig. 2)^56^ and by at least one member of the NADPH oxidase (NOX) family^57^. Ero1 uses the oxidative power of molecular oxygen to initiate redox relays which ultimately lead to disulfide bond generation in newly synthesized, folded proteins^58^, thus identifying oxidative protein folding as a major cellular ROS source^59^. Important partners in this relay are the members of the protein disulfide isomerase (PDI) family, folding assistants with thioredoxin (Trx)-like domains that catalyze the formation or isomerization of inter- and intra-molecular disulfide bonds into ER proteins^60,61^. Reduced PDI, the product of disulfide bond formation, retains catalytic functions as an isomerase, but also as an enzyme needed to eliminate misfolded proteins from the ER via its reducing power, potentially in collaboration with another ER oxidoreductase, ERdj5^62,63^. ER peroxidases, such as peroxiredoxin 4 (Prx4), as well as the glutathione peroxidases GPx7 and GPx8 scavenge luminal H_2_O_2_ arising from Ero1 and NOX, and normally prevent H_2_O_2_ leakage from the ER^49,64–66^ (Fig. 2). As an alternative pathway, Prx4 can convert H_2_O_2_ to disulfide bonds on nascent polypeptides^66^. This role of ROS may also rely on a chemical oxidation of PDI family proteins^67^ and on increased levels of oxidized glutathione (GSSG) in the presence of ROS^68^. It is currently not known whether an accumulation of mitochondrial or peroxisomal ROS could similarly mediate the catalytic formation of disulfide bonds.

Persistent ER stress increases production of ROS at the ER^69^, suggesting that the accumulation of unfolded proteins leads to redox-amplified imbalances in the Ero1/PDI electron flow^70^. These may be counteracted by an influx of reduced glutathione (GSH)^71–73^. In that context, it is important to note the role of the cytosolic redox environment, since the inhibition of cytosolic thioredoxin reductase 1 influences successful ER disulfide bond formation^74^. The recent advent of redox probes for the cytosol, and the organelles of the redox triangle^75–77^ will facilitate further investigation of these questions.

## Targets of oxidative stress in mitochondria, peroxisomes and the ER

The presence of antioxidant enzymes within mitochondria (SOD), peroxisomes (catalase) and the ER (peroxiredoxin 4 and glutathione peroxidase 7 and 8) initially suggested that ROS are toxic byproducts^78^. Thus, they were thought to promote aging: consistent with this idea, the cytosol of C. elegans cells becomes more oxidizing with age, while the ER becomes more reducing^79^. However, it is now firmly established that H_2_O_2_ acts as a key second messenger in cell growth or differentiation. Accordingly, dedicated transporters shuttle H_2_O_2_ between organelles, like aquaporins on the ER and the plasma membrane^80,81^. For instance, aquaporin-8 localizes to the ER^82,83^ or mitochondria^84^. Moreover, peroxisomes contain Pex11, which shows homology to transient receptor potential (TRPM) ion channels and transports beta-oxidation metabolites^85^, but a bona fide peroxisomal channel for ROS is currently not known^86,87^.

**Fig. 3:**
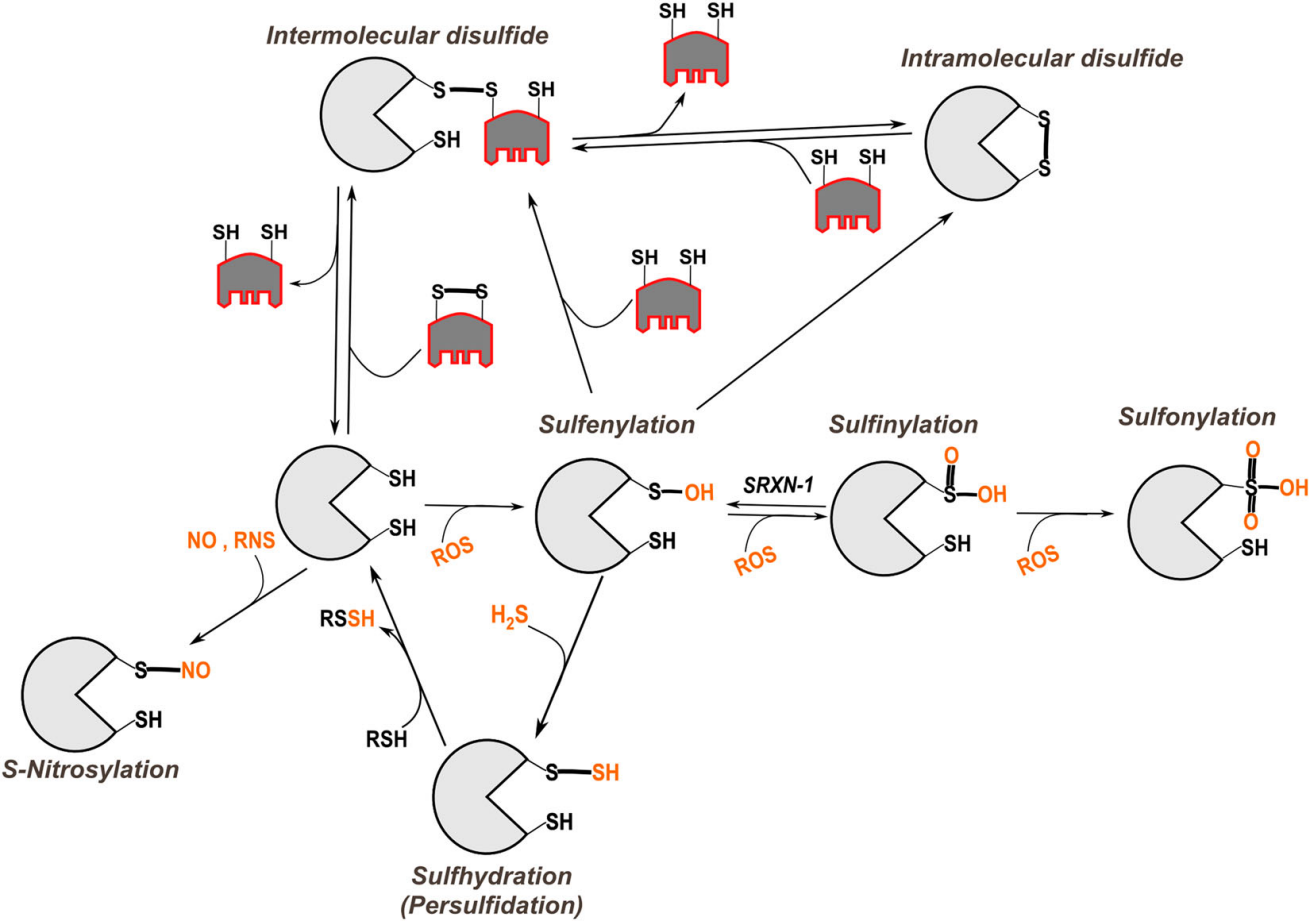
Overview of redox post-translational modifications of cysteines Oxidation by ROS (like H_2_O_2_) initially leads to sulfenylation (SOH). Sulfenylated cysteine can additionally react with ROS leading first to sulfinylation (SO_2_H) and then to sulfonylation (SO_3_H). While sulfonylation is so far considered irreversible, sulfinylation can be reversed through the catalytic activity of the cytoplasmic enzyme sulfiredoxin-1 (SRXN-1). Many reactions can lead to disulfide bond formation: (i) intermolecular disulfide bonds can be formed with another protein or low molecular weight thiols (glutathione for example), (ii) intramolecular disulfide bonds are often inserted into a reduced protein by disulfide exchange (via formation of mixed disulfides) with GSSG or another oxidized protein (e.g., PDI) or through reaction of the relative instable sulfenylated cysteine. Please note that reactions involving thiol groups (SH) implies the formation of thiolate (_-_S^-^) through deprotonation and so are strongly dependent on the local pKa

Owing to their chemical properties, ROS can modify target proteins, changing their conformation or activity. Reversible redox modifications provide a powerful signaling network, not unlike phosphorylation^88^. For instance, cysteine residues can adopt numerous oxidation states (SS, SOH, SO_2_H, SO_3_H, SNO, SSH etc.), offering a large panel of physiological protein redox modifications (Fig. 3)^89,90^.

ROS play important roles at the ER, where their production activates the unfolded protein response (UPR), accompanied by the inactivating sulfhydration of protein tyrosine phosphatase 1B (PTP1B) that results in the increased phosphorylation and, hence, activation of PERK^91^. Another example is ROS-mediated sulfenylation, which shifts the activity of the UPR kinase Ire1 from ER stress signaling to activating Nrf2^92^. At the level of mitochondria, ROS lead to oxidation of components of the respiratory chain^43^ or sulfenylation of uncoupling protein-1, resulting in increased thermogenesis^93^. Within peroxisomes or in the cytoplasm, peroxisome-derived H_2_O_2_ oxidizes Prx2^94,95^, which leads to the degradation of this ROS scavenger ^96^.

Cysteine oxidation can also engender intra- or inter-molecular disulfide bonds that stabilize or regulate the tertiary and quaternary structures of proteins. This is shown for example with the Nrf2 transcription factor, where H_2_O_2_ mediates the formation of an activating disulfide bond within its regulatory partner KEAP1^97,98^. Enzymes mediating ER oxidative protein folding such as BiP/Grp78 are also targets of ROS modifications that serve to modulate their chaperoning function^67,99^.

Another group of redox-controlled modifications on cysteines are glutathionylations. ER proteins are frequent targets of this modification^100^, including many important regulators of the interactions with mitochondria like calnexin and SERCA Ca^2+^ pumps^101^. This modification results in the activation of SERCA^102^. At the level of mitochondria, ROS can trigger glutathionylation of the pyruvate dehydrogenase complex, another source of mitochondrial ROS, which further increases oxidative stress^103^. Moreover, mitofusins can undergo redox-dependent disulfide bond formation, leading to their dimerization and potentially glutathionylation, which promotes mitochondrial fusion aiming to mitigate oxidative stress-mediated damage on oxidative phosphorylation^104^. At the same time, mitochondrial Ca^2+^ uniporter glutathionylation promotes activation of mitochondrial Ca^2+^ import, resulting in the activation of oxidative phosphorylation, but also increasing apoptosis^105^. Counteracting these modifications is the mitochondrial protease Lon, which degrades ROS-modified mitochondrial proteins^106^. Interestingly, peroxisomes also contain their own Lon protease (pLon), suggesting similar mechanisms may be at play in this organelle as well^107^. This peroxisomal protease can interact with enzymes involved in beta-oxidation and mediate their activation^108^, but peroxisomal Lon also plays a role in the sorting of PTS1 proteins and can act as a chaperone^109^.

The role of ROS also has an inter-organellar dimension: for example, ROS leaking from mitochondria interact with glyceraldehyde 3-phosphate dehydrogenase (GAPDH), which inactivates the active cysteine of this enzyme via formation of a disulfide bond, and hence blocks glycolysis^110^. Moreover, mitochondrial ROS also dictate the cellular disulfide proteome^111^, suggesting that mitochondrial ROS cross-influence in particular the ER. Similarly, increased peroxisomal ROS production can leak to the cytosol, where it can oxidize important signaling molecules, including NF-kB and PTEN^94^. ROS leak could also occur across aquaporin-8, the ER ROS pore^112^. Together, these currently preliminary observations raise the possibility that redox signaling functions in interorganellar and intercellular ways between the “redox factories” of eukaryotic cells: mitochondria, peroxisomes and the ER.

## ER-mitochondria redox signaling via mitochondria-associated membranes (MAMs)

The ER is a multitask organelle that coordinates the “validation” or degradation of secretory proteins, but also synthesizes lipids and is the main intracellular Ca^2+^ store with a luminal concentration close to the extracellular milieu (0.5–1 mM)^113,114^. Since the mid 20th century, researchers had noticed that some ER regions were in close proximity with mitochondria^5^ and also that mitochondria isolated by classical subfractionation were tightly associated to ER tubules and vice versa^115,116^. In 1990, enzymes catalyzing the synthesis of phosphatidylserine (PS) and phosphatidylcholine (PC) were shown to localize to such ER-mitochondria contact sites, called mitochondria-associated membranes (MAMs)^9^. A combinatorial approach using light microscopy techniques and Ca^2+^ probes showed that mitochondria-ER contacts (MERCs) also use juxtapositions between ER Ca^2+^ release channels and mitochondrial Ca^2+^ uniporter (MCU) to shuttle this ion from one organelle to the other^117^.

Given that mitochondria and ER are two intracellular redox hubs, it would come as no surprise if redox-related communication between the two occurred as well. Consistent with this hypothesis, the Hajnoczky lab recently managed to target a H_2_O_2_-specific fluorescent probe to MAMs^31,118^. Using this new tool, they detected the Ca^2+^-dependent generation of redox nanodomains at the level of ER-mitochondria contact sites. These nanodomains were formed by mitochondrial H_2_O_2_ release upon IP_3_R-mediated Ca^2+^ release at the interface between both organelles that caused K^+^ and water influx into the mitochondrial matrix. The ensuing mitochondrial matrix swelling decreased the mitochondrial cristae volume, provoking the release of their H_2_O_2_ content.

At this point, it is unclear what the consequences of this local ROS accumulation at the ER-mitochondria MCS are, but ryanodine receptors^119^, IP_3_Rs and SERCA Ca^2+^ pumps are known targets of ROS, which alter their activity^120^. The readout of MAM-localized ROS is expected to result in SERCA inactivation^121,122^ and IP_3_R activation^123^, thus leading to a feed-forward loop for ER-mitochondria Ca^2+^ flux. The ER oxidoreductases ERdj5 and SEPN1 counteract this accelerated ion flux, and activate SERCA by reducing its oxidation^16,124^. Conversely, ERp44 interacts with IP_3_R1 under reducing conditions to inhibit ER Ca^2+^ release^125^. Opposing these protective functions, other oxidoreductases act to boost ER-mitochondria Ca^2+^ crosstalk. Two prominent examples are Ero1α and TMX1. Ero1α acts on IP_3_Rs, potentially competing with ERp44^15^, increasing Ca^2+^ release from the ER. Moreover, Ero1α reduces mitochondrial Ca^2+^ uptake^14^. Conversely, TMX1 inactivates SERCA^126^, thus reducing Ca^2+^ uptake from the cytosol. A more ambiguous role is played by GPx8, which inactivates SERCA, but due to the reduction of the ER Ca^2+^ reservoir, also reduces ER-mitochondria Ca^2+^ flux^11^. Through their combined activities, these oxidoreductases, previously considered exclusively part of the ER folding machinery, form a redoxosome at the MAM, where they predominantly localize^10,11,14,126^. Such a redoxosome would be a multimeric, multiorganellar protein complex that mediates or controls MCS formation in a redox-specific manner. It is currently unclear whether any ROS production from Ero1α itself influences the redoxosome from the MAM.

Bax inhibitor 1 (BI-1) localizes to MAM as well^127^ and reduces ER ROS in a heme oxygenase 1-dependent manner, suggesting the ER has redox-controls that go beyond the GPx proteins^128^. In addition, BI-1 has further roles for the ER redox due to its association with the NADPH-dependent cytochrome P450 reductase^129^.

Less is known about redox-controlled proteins that localize to the mitochondrial part of MCS. One example is p66Shc, which localizes to the IMS and partially to MAM, particularly in cells stressed from UV radiation or H_2_O_2_^130,131^. At the MAM, p66Shc stimulates ROS production via interference with oxidative phosphorylation, and influences apoptotic pathways^132^. This occurs following the phosphorylation by protein kinase C beta upon a shift of cellular redox to oxidizing conditions^133^. p66Shc is not only a thiol-reactive molecule^134^, but it also represses the expression of antioxidant proteins such as SOD^135^. Further connections between the IMS and MAM redox signaling are based on Mia40/CHCHD4, which can interact with MICU1^136^. Upon interaction with Mia40, this key regulator of MCU forms a disulfide bond with its sister protein MICU2 and thus regulates Ca^2+^ uptake.

A potential link of MAM redox reactions exists with the machinery that mediates mitochondrial dynamics. GSSG promotes mitochondrial fusion, priming glutathionylation of mitochondrial mitofusins, in the case of mitofusin-2 on cysteine 684^137^, thought to be located on the cytoplasmic portion of mitochondria^138^. While GSH causes the reduction of this cysteine and reduces electron flux on the oxidative phosphorylation chain^104^, ER-derived ROS could have the opposite effect, thus outlining a pathway that could directly connect ER oxidative protein folding with mitochondrial dynamics^139^. In contrast, arrest of oxidative phosphorylation by hypoxia triggers the outer mitochondrial membrane protein FUNDC1 to exchange MAM-localized calnexin for Drp1 and thus, to mediate mitochondria fission^140^. Remarkably, these findings indicate that oxidative phosphorylation is monitored at the level of the MAM using ROS output by ER chaperones to elicit alterations in mitochondrial structure.

Redox imbalances in the ER have consequences inside mitochondria as well. This is nicely demonstrated by the mitochondrial protease Lon whose expression increases upon ER stress^141^ and an increase of mitochondrial respiration during ER stress^142^. Conversely, mitochondrial ROS exacerbate ER stress^143^, suggesting there is a feedback loop that reinforces ROS production in both organelles^144^. In yeast, this mechanism has recently been shown to depend on the ER-resident NADPH oxidase Yno1p that ramps up ER ROS production upon loss of cytochrome c oxidase. This finding led to the surprising identification of the ER as a prime source of ROS following problems at the level of oxidative phosphorylation^145^. Underscoring the importance of the ER as a ROS producer and sentinel for redox imbalances, the oxygen-sensing HIF1α transcription factor localizes to the ER, where it undergoes a stabilizing Fenton reaction that depends on pO_2_ and subsequently enters the nucleus to trigger the hypoxia response^146^. At the moment, it is unclear whether these important redox communication pathways between the ER and mitochondria require a functional MAM.

## Peroxisomes contribute to the redoxosome

Intriguingly, peroxisomes localize to triple contact sites with the ER and mitochondria in yeast^147^, formed via the interaction of Pex11 with the ER-mitochondria encounter structure (ERMES)^148^. These findings support the idea that they are part of the cellular redoxosome, forming a “redox triangle”. Redox activities in peroxisomes influence mitochondrial redox^33^. Peroxisomes are connected to the ER and mitochondria in many ways: the connection to the ER is obvious, given the de novo biogenesis of peroxisomes originates at the ER^149^, especially when considering mammalian cells^150^, where ER structures can wrap around peroxisomes^151,152^. Both mitochondrial and ER structures are compromised in the absence of peroxisomal activity in a Pex5 knockout model^153^. These abnormalities include the swelling of mitochondria and increased ROS production by the oxidative phosphorylation electron relay^154^. In liver cells, peroxisome deficiency from a Pex2 knockout, likely resulting in increases of local ROS levels, leads to ER stress and PERK activation^155^. In contrast, plant peroxisomal catalase depends on Ca^2+^ import into the organelle^156^, which is tied to the availability of cytosolic (and therefore presumably ER-released) Ca^2+^ ^157^.

Furthermore, the production of peroxisomal ROS can trigger mitochondrial apoptosis pathways, suggesting ROS are transmitted over to this organelle^158^. Indeed, ROS are thought to readily cross peroxisomal and mitochondrial membranes^159,160^. Such functional connections are not surprising, given that beta-oxidation of fatty acids can be initiated in peroxisomes, but must be concluded in mitochondria^161^. The shuttling of the beta-oxidation intermediates may occur at physical contacts between the two organelles^162^ or potentially via as of yet uncharacterized bi-directional vesicular trafficking^163^, likely involving carnitine acetyl transferases^164^. Interestingly, mitochondria and peroxisomes share key components of their division machinery^165^, raising the possibility for such a currently hypothetical mechanism.

The requirement to replenish NAD^+^ needed for beta-oxidation also suggests the existence of additional redox shuttle systems between the peroxisome and mitochondria involving malate and aspartate in yeast^166^, but their identity is currently not known in mammalian cells^1^. At peroxisome-mitochondria MCS, further redox shuttles may promote the reduction of NADP to NADPH^167^. The redox-related link between peroxisomes and mitochondria is further illustrated by the improvement of mitochondrial functioning upon increased amounts of peroxisomal catalase or peroxisomal beta-oxidation^168,169^. However, these effects may be connected to the simple increase of ROS, concomitant with an increased level of oxidative stress upon peroxisome interference^154^.

Interestingly, particularly mammalian mitochondria might communicate with peroxisomes in the inverse direction via the generation of mitochondrial pre-peroxisomes carrying peroxisomal proteins including Pex3^170^, Pex12, Pex13, to name but a few examples, that fuse with ER peroxisomal precursors^171^. At this point, it is not clear whether these structures mediate redox crosstalk or whether they are ROS-sensitive, although their existence opens up such a possibility. As mentioned earlier, it is also not clear whether an inverse trafficking mechanism exists.

## Conclusion and perspectives

The 21st century has witnessed the molecular characterization of membrane contact sites (MCS) that regulate the intracellular spatial organization of the compartments in eukaryotic cells. However, we have likely only seen the tip of the iceberg in terms of their full functionality. Thus, much has to be learned about the molecular events that mediate MCS crosstalk and how it is regulated. The molecular targets of the tight MAM-ER redox and Ca^2+^ signaling interplays remain largely uncharacterized. Further integration of redox signaling will also have to take into account the seemingly equal importance of peroxisomes to form a “redox triangle” that could depend on a multiprotein complex we propose to call the MCS “redoxosome”. The known dysfunctions of all three components of the cellular redox triangle formed by mitochondria, peroxisomes and the ER during aging could really all arise from a dysfunctional redoxosome. The existence of the redoxosome could allow the cell to balance the production and elimination of ROS within the ER, the mitochondria and peroxisomes and adjust it to growth and stress conditions. This makes sense, since the upstream needs and downstream interactions of the three organelles are tightly connected. In fact, it is today unthinkable to imagine the functioning of any of the three organelles as a single unit, without the interaction with their redox partners. The best examples to illustrate these links are the shuttling of beta-oxidation intermediates between peroxisomes and mitochondria and the induction of mitochondrial proteins following ER stress.

Future experiments will have to test our proposal. Such experiments could involve the interference with organelle tethering, for instance by knocking out tethers that we postulate would be required for the proper functioning of the redoxosome. At the moment, such approaches can lead to results, which are difficult to interpret, as shows the example of mitofusin-2^172,173^. One reason for such difficulties could be that the interference with tethers will affect functional interactions at MCS, including the redoxosome.

Currently, the identity of tethers is being elucidated not just for the ER and mitochondria^174^, but also for peroxisomes: pioneering studies in yeast have used peroxisome inheritance to identify a Pex3p-Inp1p complex that attaches them to the ER^175^. Using more recent BioID approaches in mammalian cells, a complex between ER-localized VAMP-associated proteins A and B (VAPA and VAPB) and with the peroxisomal membrane protein acyl-CoA binding domain containing 5 (ACBD5) was shown to act as a tether as well^37,38^. Less is known about peroxisome–mitochondria tethers, but a recent publication has characterized a complex between Pex11 and ERMES as a putative tether^148^.

To solve these questions enhanced efforts will be required to characterize biochemically and anatomically the MCS proteomes in time and space^176^. The evidence presented in this review that MCS may critically depend on multiple organelles interacting with each other presents both an unexpected difficulty for this task, but also a chance for exciting discoveries in future.
